# Alcohol minimum unit pricing and people experiencing homelessness: A qualitative study of stakeholders' perspectives and experiences

**DOI:** 10.1111/dar.13548

**Published:** 2022-09-28

**Authors:** Elena D. Dimova, Heather Strachan, Sarah Johnsen, Carol Emslie, Martin Whiteford, Robert Rush, Iain Smith, Tim Stockwell, Anne Whittaker, Lawrie Elliott

**Affiliations:** ^1^ Glasgow Caledonian University Scotland UK; ^2^ Heriot‐Watt University Scotland UK; ^3^ Independent Consultant Edinburgh UK; ^4^ NHS Forth Valley Scotland UK; ^5^ Canadian Institute for Substance Use Research University of Victoria Victoria Canada; ^6^ Nursing, Midwifery and Allied Health Professions Research Unit, Faculty of Health Sciences and Sport University of Stirling Scotland UK

**Keywords:** alcohol, health policy, homeless people, minimum unit pricing, Scotland

## Abstract

**Introduction:**

Minimum unit pricing (MUP) may reduce harmful drinking in the general population, but there is little evidence regarding its impact on marginalised groups. Our study is the first to explore the perceptions of MUP among stakeholders working with people experiencing homelessness following its introduction in Scotland in May 2018.

**Methods:**

Qualitative semi‐structured interviews were conducted with 41 professional stakeholders from statutory and third sector organisations across Scotland. We explored their views on MUP and its impact on people experiencing homelessness, service provision and implications for policy. Data were analysed using thematic analysis.

**Results:**

Participants suggested that the introduction of MUP in Scotland had negligible if any discernible impact on people experiencing homelessness and services that support them. Most service providers felt insufficiently informed about MUP prior to its implementation. Participants reported that where consequences for these populations were evident, they were primarily anticipated although some groups were negatively affected. People experiencing homelessness have complex needs in addition to alcohol addiction, and changes in the way services work need to be considered in future MUP‐related discussions.

**Discussion and Conclusions:**

This study suggests that despite initial concerns about potential unintended consequences of MUP, many of these did not materialise to the levels anticipated. As a population‐level health policy, MUP is likely to have little beneficial impact on people experiencing homelessness without the provision of support to address their alcohol use and complex needs. The additional needs of certain groups (e.g., people with no recourse to public funds) need to be considered.

**Key Points**

- This is the first study to explore the experiences and perceptions of MUP among stakeholders who work with people experiencing homelessness, such as service providers, following its introduction in Scotland in May 2018.
- Service providers reported little impact of MUP on these populations and their work with them.
- Many service providers reported feeling insufficiently informed about the implementation of MUP and so missed opportunities to use it to initiate conversations with service users regarding reducing alcohol‐related harms.
- Concerns about potential harms from MUP rarely materialised and consequences of MUP among people experiencing homelessness were mostly intended (e.g., reduction in alcohol consumption; switching away from cheap strong cider).
- According to service providers, unintended consequences were experienced among some people with no recourse to public funds.
- Countries considering MUP should support stakeholders prior to implementation so that they are equipped to capitalise on potential benefits as well as mitigate any harms.

## 1 INTRODUCTION

Research evidence suggests that price‐based alcohol policy interventions, such as minimum unit pricing (MUP), reduce alcohol consumption and alcohol‐related morbidity and mortality [1, 2, 3, 4, 5]. Unlike other pricing policies (e.g., social referencing pricing, which is determined by the volume and type of alcohol) [6], MUP sets a minimum unit price for all alcoholic drinks. Its intention is to reduce the affordability of high‐strength, low‐cost alcohol and subsequently alcohol‐related harms [7]. In 2018, the Scottish Government introduced a MUP policy, setting the price for alcohol at £0.50 per unit (1 unit = 8 g ethanol). This legislation contains a review clause, meaning it will expire 5 years after implementation unless the Scottish parliament votes for it to continue [8, 9].

Pricing policies have been widely supported internationally [10, 11, 12] and already operate in Canada [13], Russia [14] and some former Soviet Union countries [15]. More recently, MUP was introduced in Australia [16], Wales [17] and the Republic of Ireland [18]. Modelling studies suggest that MUP will be most effective in reducing alcohol consumption and alcohol‐related harms among heavy and harmful drinkers, particularly those with low income [19, 20]. This has also been shown in practice in British Columbia, Canada, in relation to other forms of alcohol pricing policies [21].

Survey research from the United Kingdom suggests that MUP may have a disproportionate effect on people who drink at harmful levels, have limited income and drink less expensive alcohol [20]. Recent modelling based on UK surveys and routine sales and health data collected before MUP, also suggests that there may be significant gains among people who drink at harmful levels and have low‐income including reductions in consumption, mortality and morbidity [22]. None of these studies explore the lived experience of MUP among people experiencing homelessness and/or street drinking or those who provide services to these groups. People experiencing homelessness, here defined as people with experience of rough sleeping, temporary or insecure accommodation [23], and people who regularly drink outdoors in public places [24], represent highly marginalised populations. Their drinking patterns differ from those of the general population in two key ways: first, the volume and types of alcohol typically consumed are especially damaging to health [25] and second, much of it is consumed in public places and is often associated with antisocial behaviour and public complaint [24, 26]. People experiencing homelessness may be susceptible to pricing policies since levels of alcohol‐related harm and alcohol use disorder among these populations are much higher compared to the rest of the population [27, 28, 29]. In the period 2020–2021, there were 30,345 adults in homeless households in Scotland and the majority of individuals affected were male and between the ages of 25 and 49 years [30].

Existing studies have looked more generally at homeless people's strategies when alcohol becomes less affordable [31, 32]. Some strategies include reducing alcohol consumption and re‐budgeting of funds [30]. Our study used a qualitative approach to explore the impact of MUP in Scotland on people experiencing homelessness and/or drink on the streets, and the services that work with them [33]. This paper focuses solely on the perspectives of key stakeholders, working strategically or directly with this population. Their perspectives are important because alcohol control policies such as MUP involve implementation and regulation across different sectors, including health, social care, housing and law enforcement. Better understanding of service providers' experiences of MUP can be used to help countries considering pricing policies to capitalise on potential benefits as well as mitigate any harms.

## 2 METHODS

We conducted semi‐structured qualitative interviews with stakeholders in Scotland after the introduction of MUP (we also interviewed people experiencing homelessness and/or street drinking; their experiences will be reported in a separate paper). Qualitative methods are appropriate for exploring the views and experiences of study participants and can identify emergent themes not considered at the research design stage [34]. MUP may lead to multiple anticipated and unanticipated outcomes in different populations and contexts, and a qualitative design allows for a more nuanced exploration of these phenomena from the perspective of different stakeholders.

### 2.1 Study steering and stakeholder groups

We convened a study steering group, which included academics in public health and representatives from charity organisations working with homeless people. The group advised on the academic, ethical and policy/service aspects of the research.

We also created a wider study stakeholder group, which included individuals with lived experience of homelessness or/and street drinking and alcohol use, representatives from the study partner organisation (Homeless Network Scotland) and from third sector organisations, policy makers and academics. The stakeholder group met at key project stages and advised on our methodological approaches, commented on interpretation of findings and helped us to formulate policy and service level recommendations based on the study findings.

### 2.2 Participants and recruitment

We used pragmatic purposive sampling through professional networks and snowball referrals. To capture a range of views and experiences, we recruited stakeholders with at least 2 years' experience of working in the field of homelessness and/or addiction and allied health and social care fields across Scotland. Potential participants were approached either by directly emailing them or by emailing managers and asking them to inform their staff of the study. The respondents were key stakeholders who work closely with homeless and street drinkers and/or were involved in the development and introduction of MUP in Scotland. They were recruited from the following sectors: third sector, including homeless, social care and substance use services (*n* = 18), health services including accident and emergency, substance use services, primary care and pharmacies (*n* = 11), government/policy makers (*n* = 4), police (*n* = 3) and local councils (*n* = 5). Participants' organisations operated in major cities in Scotland (Edinburgh, *n* = 9; Glasgow, *n* = 12), in rural areas (Inverness, *n* = 4) and at a national level (*n* = 16). Respondents' roles included: manager (*n* = 10), team leader (*n* = 7), health‐care professional (e.g., nurse, psychiatrist, pharmacist; *n* = 10), social worker/support worker/outreach worker (*n* = 4), police officer (*n* = 3), policy maker/public health (*n* = 3), housing officer (*n* = 2) and other roles (*n* = 2).

### 2.3 Data collection

We conducted 38 interviews with 41 participants (*n* = 36 one‐to‐one, *n* = 1 with two stakeholders, *n* = 1 with three stakeholders) between November 2020 and April 2021. Due to the COVID‐19 pandemic, the majority of interviews were conducted remotely via phone (*n* = 5) or a video call (*n* = 33). Interview duration varied from 21 to 78 min, with most lasting 40 to 50 min. A study information leaflet and consent form were given to participants and informed consent was recorded prior to the interview. To maintain anonymity, each respondent was assigned a unique ID number.

The interviews followed a semi‐structured topic guide, developed through conversations between the study team and the partner organisation (Homeless Network Scotland), with feedback from the study steering group and a wider stakeholder group. The topic guide contained open‐ended questions on respondents' views and experiences of MUP, with a focus on any impact on people experiencing homelessness and street drinking, and services. We also explored implications for policy practice and service provision. Other topics included the service and policy landscape and observed effects on service users that may be relevant to MUP, for example, wellbeing, housing, social and economic hardship.

### 2.4 Data analysis

With permission from participants, interviews were audio recorded and then transcribed by professional transcribers. Transcripts were checked for accuracy by a member of the research team, who also removed any identifying information.

Our analysis was informed by a social constructionist epistemology, which views the world as having multiple systems of understanding that occur through social and cultural experiences, which in turn are largely influenced by language [35, 36]. Language is viewed as active and constructive, as shared social meanings arise primarily through language [36].

Data were analysed using thematic analysis [37, 38], facilitated by NVivo v12 software. After initial familiarisation, a coding framework was developed by one researcher (HS) based on the interview schedule and emergent topics, and discussed with the wider team. This initial coding framework was structured into broad categories such as participants' awareness of MUP, impact of MUP on services, observed impact of MUP on people experiencing homelessness and street drinking. The transcripts were then coded line‐by‐line by one researcher (HS) using a mixture of deductive coding based on the coding framework and an inductive, open‐coding approach based on new emerging issues. A second researcher (EDD) independently double coded 20% of the transcripts and there was no discrepancy between coders. After this, data were charted into framework matrices according to emerging themes and sub‐themes. Finally, similarities and differences between the data were explored and discussed at weekly research team meetings. The final over‐arching themes, which were underpinned by multiple sub‐themes, include: ‘Impact of MUP on services’, ‘Impact of MUP on people experiencing homelessness and street drinking’, ‘Relevance of the wider context for people experiencing homelessness’ and ‘Future policies and service development’. In the sections below, we use the term ‘policy makers’ to refer to respondents who were involved in the development of MUP and ‘service providers’ to refer to those who work or interact with people experiencing homelessness and street drinking in either management or frontline roles (e.g., homeless shelter managers, health‐ and social‐care workers, police officers).

## 3 RESULTS

In this paper, we present participants' views and experiences of MUP. First, we focus on stakeholders' expectations before the introduction of MUP and their experiences of how MUP impacted on services. Then, we explore participants' views on how MUP affected their clients (i.e., people experiencing homelessness and street drinking). Finally, we draw attention to the wider context in which MUP operates and consider participants' suggestions for future policies or service change.

### 3.1 Impact of MUP on services

Most participants supported MUP as a public health policy. The level of preparation before the introduction of MUP varied across services, although it rarely went beyond receiving briefing information and discussions among staff. Accounts suggested that after MUP was introduced, immediate impacts on services were seldom observed.

#### 3.1.1 Expectations of MUP: ‘I understand the reasoning behind it, but I was sceptical’

Most participants understood the rationale behind MUP and supported it as a public health policy. However, before its introduction, service providers working directly with people experiencing homelessness had concerns about potential negative consequences, such as people prioritising alcohol over basic needs or using other substances:

‘*Concerns were expressed about what might that* [MUP] *mean* [for people experiencing homelessness] *and their use not just of alcohol but potentially of other more dangerous substances, potentially*’. (SP19 Manager, Third sector alcohol organisation)

In some cases, concern about the potential negative impact of MUP on people experiencing homelessness appeared to contribute to scepticism about the likely efficacy of MUP:

‘*I understand the reasoning behind it, but I was sceptical as to whether it was even going to work, you know? Just based on the fact that the population that I work with, people will beg, borrow, steal, to get their drug of choice*’. (SP10 Pharmacist)

#### 3.1.2 Service preparation: ‘I don't think we made any particular preparations for it’

The level of preparation before the introduction of MUP varied across services. Staff within different organisations had discussed how it might affect service users: ‘there were those discussions: are more people going to come into treatment?’ (SP30 Addictions nurse). Some organisations received information about MUP. For example, the Scottish Government worked with Alcohol and Drug Partnerships (i.e., multi‐agency groups tasked by the Scottish Government with tackling alcohol and drug issues in a particular area) to ensure ‘staff were briefed on what may come to pass as a result of a price change, which could be significant for some of the people in their communities, so that they were able to communicate and engage with those who may be seeking help’(SP04 Policy maker).

However, many participants reported that organisations did not engage in formal preparations to help mitigate potential consequences:

*‘Yeah, I think certainly what I learnt* [about MUP] *was more on the media … the generalised information that was out there. There were no specific strategies, that I'm aware of, in relation to it getting rolled out, as in pros, cons, what to look for, what not to look for, any of that*’. (SP21 Police)

One third sector organisation reported setting up ‘contingency plans’ in anticipation of people who drink heavily reducing/stopping drinking after MUP, but this level of preparation was atypical:

‘*So there was a kind of whole huge “how will this affect people, how are we going to support people if they're maybe suffering from DTs* [delirium tremens]*, if they're heavy drinkers and they're now having to withdraw* [from alcohol]*.” So there were kind of contingency plans discussed and put in place as to how we best support people*’. (SP38 Team Leader, Third sector homelessness organisation)

#### 3.1.3 Impact on services: ‘It was a useful time for us to have a conversation with people’

Accounts suggested that after MUP was introduced, immediate impacts on services were seldom observed. Several participants across police, statutory services and third sector reported they ‘didn't see any change’ (SP15 Psychiatrist) in the way services were delivered. For example, the service provider (SP38), who reported plans to support people experiencing withdrawal symptoms, found that the expected increase in hospital admissions did not materialise:

‘*To be honest, we didn't really see it because I think it affected drinks like cider. It didn't really affect spirits, because they're already expensive. So I think there was a lot of talk about it. I don't think we really seen the impact*’. (SP38 Team Leader, Third sector homelessness organisation)

Another participant, working for statutory services, reported an increase in hospital admissions for withdrawal symptoms:

*‘Yeah, so they're going to feed the addiction regardless of what they have to sacrifice to do that. And if they don't, then they become unwell. They become unwell, go into withdrawal, they end up in hospital and it starts all over again when they come into the community hospital. They go and do the same thing. We have seen quite a lot more hospital admissions in regard to withdrawals I have to say*’. (SP16 Addictions nurse)

Despite initial concerns about people experiencing homelessness prioritising alcohol over food and other necessities, this was not commonly reported. Some service providers reported an increase in the use of food banks and drop‐in centres that provide free lunches. However, this was sometimes hard to disentangle in people's accounts from the more recent effect of the COVID‐19 pandemic:

‘*It's* [use of foodbanks] *probably been really, really skewed obviously in the last year with the COVID thing* […] *But yeah, thinking before then, I think we probably did see an initial upswing in people coming in, and being motivated to go along to drop ins and, you know, free lunch type set ups*’. (SP26 Housing officer, Local council)

According to a few third sector service providers, MUP was seen as an opportunity to discuss alcohol‐related harm reduction strategies with people experiencing homelessness and street drinking presenting at services:

‘*So, people were saying to us, “Oh well, we'll probably not' buy that cider anymore.” And they'll switch to something else. So, it was a useful time for us to have a conversation with people about it and suggest an alternative*’. (SP05 Manager, Third sector homelessness organisation)

### 3.2 Impact of MUP on people experiencing homelessness

The impact of MUP reported most frequently by service providers was service users switching from cheap, strong ciders to spirits, wine or strong beer. A few service providers said that some people experiencing homelessness and street drinking supplemented alcohol beverages with illicit drugs in addition to isolated examples of non‐beverage alcohol use.

#### 3.2.1 Intended consequences: Reduction in alcohol consumption, switch away from strong cider, help seeking

As MUP targeted high‐strength low‐cost alcohol, it was not surprising that this was felt most keenly among people who consumed cheap, high alcohol content ciders (e.g., 7.5% alcohol by volume). Service providers believed that a switch from these ciders to others that were less ‘potent’ may be beneficial for people's health:

‘*The stronger cider is obviously gone up enormously in price, so … it's probably been beneficial for their health, a lot of them have gone from that really poisonous, strong cider, this X type stuff, they've gone down to Y, which is a 5% regular, apple‐based cider, so I would say it's improved their health in many ways*’. (SP03 Manager, Third sector homelessness organisation)

In some cases, the increased cost of alcohol was presented as a motivating factor for a reduction in alcohol consumption and for help‐seeking:

‘*But there are a couple of folk, that actually came and for the first time in forty years came and asked for help because they could no longer afford to sit and drink cider outside the cathedral anymore*’. (SP13 Social Worker, Local council)

While some people switched from high‐strength to lower‐strength ciders, others opted for stronger beverages, such as vodka and fortified wines. Although MUP may have changed what people experiencing homelessness drank, some practitioners thought that it did not necessarily change the amount consumed:

‘*And there was like subtle manoeuvrings of how they drank. But not, there's no massive changes etc. As I say, somebody who would rather drink cider could get a half bottle of vodka instead, and a mixer or take it with water, etc*’. (SP08 Team Lead, Third sector homelessness organisation).

However, a few service providers expressed concerns about people learning to calibrate the consumption of spirits when they were used to drinking cider, meaning they may have increased the units they drank as ‘they would drink the same amount of the spirit, which obviously is far stronger’ (SP31 Manager, Third sector homelessness organisation).

#### 3.2.2 Unintended consequences—Negative health consequences, increase in drug use among those already using, non‐beverage alcohol

Some participants thought that MUP resulted in a harmful switch to spirit use, which in some cases led to acute intoxication. Examples based on experience include increased seizures, falls, head injuries or gastric bleeds:

‘*But as soon as Minimum Unit Pricing came in* […] *I then went through, dealing with guys who were in a permanent fog, to dealing with the people who were having internal bleeds, you know? Head injuries from falling down the stairs. Things got a hell of a lot more dangerous overnight, basically for certain people, you know? Mostly the people who could build a raft out of the three‐litre cider bottles in their houses*’. (SP13 Social worker, Local council)

Some service providers thought there had been an increase in the use of illicit drugs (e.g., street Valium, benzos) because of MUP; mainly to use alongside alcohol rather than replacement for alcohol. While some participants attributed this to MUP, others believed it was greatly influenced by the availability of low‐priced street drugs:

‘*But that whole shifting use* [to illicit drugs] *is bigger than MUP, I mean, even in terms of the accessibility and the cost of drugs. Even the consumption, if you've got to drink three litres of cider to kind of make you feel okay, but actually a couple of these wee tablets, over with a drink of water, can have the same effect… And I don't know that the shift has been all to do with MUP*’. (SP22 Manager, Third sector homelessness organisation)

A few participants had heard about people drinking non‐beverage alcohol (i.e., hand sanitisers) and some encountered this in reports from their clients after the introduction of MUP:

‘*And when minimum unit pricing came in, we started to see people drinking non‐beverage alcohol, and mostly hand sanitiser*.’ (SP31 Manager, Third sector homelessness organisation).

Another participant reported that a team they worked with in their homelessness service had expressed significant concern that drinking hand sanitiser was an issue for people ‘who possibly didn't have recourse to public funds’. Another participant reported their experience with a group of men from the Polish community:

‘*…. the group of Polish men who've used our services for a long time, alcohol dependency is a big issue. And when minimum unit pricing came in, we started to see people drinking non‐beverage alcohol, and mostly hand sanitiser* […] *And they would mix it. They called it “macumba.” They would mix it with lemonade*’. (SP31 Manager, Third sector homelessness organisation).

Several participants believed service users would prioritise the purchase of alcohol over necessities such as food, leading them further into poverty or poor health because of these actions. While the prioritisation of alcohol was a general pattern for some service users, one participant noted a big increase in request for food bank referrals and supermarket vouchers saying they were ‘ten times busier’ and attributed this to the need to buy alcohol around the time of MUP. Another participant reported an initial increase in service users attending a drop‐in centre for ‘a free lunch’, which they perceived was linked to MUP although recognised it could also be attributed to COVID‐19:

‘*I think we saw people relying more on those, initially. It's probably been really, really skewed obviously in the last year with the COVID thing and, you know, that's been the main driver for whether people have got enough to get by on, food‐wise. But yeah, thinking before then, I think we probably did see an initial upswing in people coming in, and being motivated to go along to drop ins and free lunch type set ups*’. (SP26 Housing Officer)

Some participants had been concerned that their clients would ‘beg more or steal more because they need alcohol’ (SP07 Outreach worker, Third sector homelessness organisation). However, changes in begging or stealing by people experiencing homelessness and street drinking following MUP were not observed by the service providers in our sample. A few participants reported ‘hearing’ that cheap alcohol (i.e., at pre‐MUP prices) was available to their clients through small independent shops or ‘contraband’ alcohol.

Some participants recognised that people experiencing homelessness, especially those without access to welfare benefits, were likely to be impacted to a greater extent by MUP because they had less money to purchase alcohol and less opportunity to access services and ‘reduce their alcohol intake’ because of the circumstances that they lived in:

‘*They* [people experiencing homelessness] *won't access services, unless they're probably directed to the services and it's very hard for people to get to services. Very hard for* [homeless] *people to know they've got appointments. It's very hard for people to attend the appointment at ten a.m. on the other side of the city. They can't do that*’. (SP28 Policy maker)

### 3.3 Relevance of the wider context for people experiencing homelessness

Policies, such as MUP, do not operate in isolation and their impact is influenced by the wider social economic and policy context. During the COVID‐19 pandemic, homeless people in Scotland were provided with accommodation in hotels [35]. Some service providers believed this mitigated the effects of MUP because people who were homeless had more disposable income:

‘*They get three square meals a day* [in the hotels]. *They've just recently put in laundry facilities for them. They don't pay any rent, so they're getting all their benefits*. […] *And right through all that, there's alcohol, you know? …. because of where they're living, the pricing of alcohol maybe not have the same negative impact on them*’. (SP17 Manager, Homelessness work, Local council)

This was countered by some who thought that COVID‐19 made it harder for people to obtain alcohol due to fewer opportunities for begging and reduced availability of alcohol outlets, the implication being that alcohol consumption would stop or reduce and if required people would seek help:

‘*We've just been discussing where people couldn't access the usual methods of getting money or getting drugs or getting their alcohol, so they've actually realised that treatment is the best option for them. I think as well, you know, the fact that the hotels were there, so people had easy access to treatment, you know?*’ (SP07 Outreach worker, Third sector homelessness organisation)

Access to disposable income, primarily through welfare benefits, and support to manage their budget, were often mentioned as important in maintaining or increasing drinking. For example, one service provider explained that their role was to ensure service users had all their appropriate benefits and that once they had paid for accommodation, food and savings, the remainder was given to them either weekly or daily. This service provider concluded that on account of people having sufficient disposable income, MUP ‘hadn't been hugely significant in this environment’ (SP03 Manager, Third sector homelessness organisation). Another service provider highlighted that MUP had impacted more on those service users who received limited benefits:

‘*There was a slight change for some of the clients who maybe don't have full benefits* […] *Those people kind of changed early on in the minimum pricing to cheaper alcohol and a slightly less percentage in alcohol. However, the other ones who have more money, more* [benefits] *didn't*’. (SP16 Addictions Nurse, Statutory service)

Finally, the personal histories and day‐to‐day difficulties experienced by people who are homeless need to be considered when exploring the effects of MUP on this population. Service providers emphasised that MUP does not address the reasons underpinning the (sometimes high level of) alcohol consumption within this population, this typically being used to cope with trauma and/or to soften the hardship of everyday life and ‘make life more bearable’ (SP32 Consultant, Statutory service):

‘*…people have got an underlying trauma, pain, whatever it is that they're trying to, cope with and manage. So, if we just whip that* [alcohol] *away from them, whcat are we putting in place?*’ (SP22 Manager, Third sector homelessness organisation)

### 3.4 Future policies and service development

MUP was recognised as just one policy of a number that were required to reduce alcohol‐related harm, and a number of observations were made about potential changes to service provision and/or MUP pricing going forward.

#### 3.4.1 Changes to policies or services

Service providers recognised the need for relevant (health, housing, social care, etc.) services to work together in a trauma‐informed way to address the complex needs of people who are homeless including problem alcohol use:

*‘I think what's not fully understood is, what needs to go alongside that type of approach* [MUP] *with certain populations, including homelessness, is that you need to give people options to be able to reduce their alcohol consumption if they want, or have the ability to go into services*’. (SP28 Policy maker).

Some participants perceived the need for a ‘whole systems approach’ that ‘involves homelessness and addiction services, and as much as everybody in partnership with the NHS and everybody else’ (SP02 Team leader) to address alcohol use and alcohol‐related harm among this population. Participants were also critical of services that had entry criteria which focused on either alcohol, drugs or mental health problems. These services needed to adopt a flexible and person‐centred approach which embraced complexity:

‘*There is no resources or services to send these people to, it's because mental health services cannot assess people who are using substances. And likewise, we can't get these people into substance misuse services because of their psychotic illness or their other mental health issues’*. (SP13 Social worker, Local council).

A few participants highlighted long waiting lists for detoxification programmes (e.g., 9–10 months) and some were supportive of managed alcohol programmes, community detoxification and continued support afterwards, for example, a rehabilitation programme:

‘*A lot of times they're* [homeless drinkers] *looking for periods of stability somewhere. They're the type of services that have actually been cut back, you know, where you'd maybe go and spend a period of time for rehab or a period of time for respite, there's literally no services left like that now*’. (SP02 Team Leader, Third sector homelessness organisation)

#### 3.4.2 Potential impact of a further increase in MUP

There was a view from a few participants that a further increase in MUP would be beneficial perceiving the ‘greater the price, the greater the gain’ (SP29 Psychiatrist). They thought that increasing MUP could lead to reduction in alcohol consumption if it were introduced alongside support for those who wished to reduce their alcohol intake. That said, several other participants expressed concerns that in the absence of alcohol treatment services, a further increase in MUP may exacerbate the unintended consequences for people experiencing homelessness and street drinking:

‘*But what is the benefit going be in raising that* [MUP] *even further? Are we just trying to get it so that people who have a low income can't afford alcohol? And is the harm then going shift to other substances? Because as we've spoke about already, you know, the root cause of why they are drinking is not going be addressed*’. (SP22 Manager, Third sector homelessness organisation)

Service providers highlighted that many people experiencing homelessness are dependent on alcohol and strategies need to be put in place for those experiencing unintended impacts of MUP:

‘*I think ‐ and I'm keen to stress, specifically for my client group, it's* [MUP] *been a bad thing, you know? Because they're people that are alcohol‐dependent, that cannot access any services or resources to address that, because they're excluded by being who they are*’. (SP13 Social worker, Local council)

## 4 DISCUSSION

This paper documents findings from the first qualitative study on stakeholders' views on the impact of MUP on people experiencing homelessness and street drinking, and the services that work with them. It adds to the portfolio of studies [39] seeking to evaluate MUP in Scotland by demonstrating that initial concerns about potential harms from MUP rarely materialised and consequences of MUP among people experiencing homelessness and street drinking were mostly intended. Stakeholders reported that unintended consequences were observed among people who are homeless and have no recourse to public funds. MUP was seen to have a negligible if any discernible impact on service provision overall. Some of the service providers in our sample saw the introduction of MUP as an opportunity to discuss drinking with their clients, but it seems that exploitation of this was limited at the time. A recent study on the impact of MUP on people who are alcohol dependent (but not homeless) using treatment services in Scotland also highlights missed opportunities for services to raise awareness of MUP and available support for people wishing to reduce drinking [40]. Given health‐care providers may feel reluctant to initiate conversations about alcohol problems more generally [41, 42], future efforts need to focus on supporting service providers to have alcohol‐related discussions and to capitalise on policy implementation as an opportunity for people experiencing homelessness and street drinking to engage with harm minimisation and/or treatment initiatives.

The current study suggests that there was a shift away from cheap, strong ciders among people experiencing homelessness and street drinking, following the introduction of MUP, which is in line with policy intentions. According to the service providers in our study, the most commonly observed changes in drinking were either reducing alcohol consumption or switching to a different type of alcohol. The theory of change guiding the overall MUP evaluation in Scotland [39] considered unintended outcomes, such as displacement of spending and substitution to non‐beverage alcohol and other drugs. Service providers in our study thought that some people experiencing homelessness and street drinking switched to spirit use following MUP, which resulted in acute intoxication and negative health effects (e.g., falls, gastric bleeds). Participants reported instances of non‐beverage alcohol use after the introduction of MUP, and this might have been particularly pronounced among people who were homeless and had no access to welfare benefits. Similarly, there were accounts of a potential increase in the use of illicit drugs (e.g., street benzodiazepines) among people experiencing homelessness and/or street drinking. The response of people with no recourse to welfare benefits to MUP, is poorly understood. Given that MUP reduces the affordability of alcohol, future research is needed to explore potential unintended effects of the policy on this population.

Our study also demonstrates that despite perceptions that people experiencing homelessness would beg or steal more to obtain alcohol, engagement in illegal activities among homeless people following MUP was not observed by the stakeholders in our sample. These findings corroborate findings of a quantitative study looking at the impact of MUP on people who are dependent on alcohol and are accessing alcohol treatment services in Scotland [40]. Although study samples are different, Holmes et al. [40] also found little evidence of negative consequences following MUP in terms of illicit drug use, crime or drinking non‐beverage alcohol. However, the stakeholders in our sample expressed concerns that a further increase in MUP without provision of additional support, may increase unintended consequences for some people experiencing homelessness and street drinking (especially those without access to welfare benefits).

Study participants emphasised that people experiencing homelessness often have complex needs in addition to any alcohol related problems. There is clear evidence showing that compared to the general population, people experiencing homelessness have more comorbidity, including long‐term physical and mental health problems [28, 43] in addition to problematic drug use [44]. Reducing alcohol‐related harm is a complex problem, requiring different complementary solutions. The stakeholders in our study expressed concerns that many statutory services focus on addressing single issues, such as substance use or mental health, and as such are not designed to engage with people that fit into more than one of these categories. They therefore echoed concerns expressed by service providers working with severely and multiply disadvantaged individuals in Scotland more generally, which note that homelessness services often end up ‘carrying the can’ given failures of other sectors to meet these individuals' needs [45]. This highlights the need for a national framework for harm reduction and funding mechanisms to allow services to collaborate more effectively.

The stakeholders in our sample also emphasised that the lack of stable housing places people at risk of a whole range of harms, including but not limited to the use of alcohol and other substances. The harms associated with rough sleeping and their impact on substance use issues, and morbidity and mortality, have long been recognised [46, 47, 48]. For policies such as MUP to be effective, wider challenges experienced by this population need to be addressed. Interest and investment in Housing First, which offers settled housing and open‐ended holistic support without preconditions regarding abstinence or engagement with treatment, has increased dramatically in recent years given evidence regarding its effectiveness for homeless people with complex needs [49, 50]. Similarly, the emergence of managed alcohol programmes, as evidence‐based alcohol harm reduction approaches specifically developed for people who experience alcohol addiction and homelessness, can also provide long‐term support for this population [51, 52, 53]. In Canada, Erickson et al. [31] found that people with housing instability and alcohol dependence, who accessed managed alcohol programmes were less likely to use illicit drugs and more likely to access treatment when they could not afford to buy alcohol.

### 4.1 Strengths and limitations

This is the first qualitative study to explore the experiences of MUP among stakeholders, working with people experiencing homelessness and street drinking in Scotland. The study limitations need to be acknowledged. First, this paper does not document the views and experiences of people experiencing homelessness and street drinking; this aspect of our study has been reported elsewhere (see Elliott et al. [33] for a brief overview). Second, participants sometimes found it difficult to disentangle the effects of MUP from the broader landscape (e.g., COVID‐19; multiple disadvantages people experiencing homelessness face; availability and price of illicit drugs). In particular, the impact of COVID‐19 needs to be acknowledged. Service responses to COVID‐19 prioritised infection prevention, which in turn disrupted the availability and accessibility of health and care services [54]. This may have exacerbated risks for marginalised populations and in many cases, the participants in our study found it difficult to disentangle whether health outcomes, such as hospital admissions for withdrawal symptoms, were driven entirely by the pandemic or MUP was a contributing factor.

## 5 CONCLUSION

The introduction of MUP in Scotland had negligible if any discernible impact on services that work with people experiencing homelessness and street drinking according to the participants in this study. Most service providers reported feeling insufficiently prepared prior to its implementation, and only a few initiated conversations with service users about the policy's potential implications. Opportunities to use the introduction of MUP to promote service users' engagement with harm reduction and/or treatment were therefore missed. Despite initial concerns about unintended consequences of MUP for people experiencing homelessness and/or street drinking, participants reported primarily intended consequences (e.g., reduction in alcohol consumption, switching to other types of alcohol). Engagement in increased begging or illegal activities among homeless people following MUP was not observed by the stakeholders in our sample. However, participants reported observations of increased acute alcohol intoxication among some people who were homeless and who switched to spirts, and increased use of illicit drugs and non‐beverage alcohol. Stakeholders highlighted that this population has complex needs in addition to alcohol addiction, and changes in the way services work need to be considered as part of future MUP‐related discussions.

## AUTHOR CONTRIBUTIONS

Elena D. Dimova: Methodology, formal analysis, investigation, data curation, writing – original draft. Heather Strachan: Formal analysis, writing – review and editing.Sarah Johnsen: Conceptualisation, funding acquisition, methodology, investigation, supervision, writing – review and editing. Carol Emslie: Conceptualisation, funding acquisition, methodology, investigation, supervision, writing – review and editing. Martin Whiteford: Methodology, investigation, writing – review and editing. Robert Rush: Conceptualisation, funding acquisition, methodology, investigation, writing – review and editing. Iain Smith: Conceptualisation, funding acquisition, methodology, investigation, writing – review and editing. Tim Stockwell: Conceptualisation, funding acquisition, methodology, writing – review and editing. Anne Whittaker: Conceptualisation, funding acquisition, methodology, writing – review and editing. Lawrie Elliott: Conceptualisation, funding acquisition, methodology, investigation, supervision, writing – review and editing.

## FUNDING INFORMATION

This study was funded by the Chief Scientist Office. Project code: HIPS/18/43.

## CONFLICT OF INTEREST

None to declare.

## ETHICS STATEMENT

Ethical approval was granted by the Nursing Department Research Ethics Committee at Glasgow Caledonian University (HLSNCH18029; initially in May 2019; followed by an amendment to conduct remote interviews due to COVID‐19 restrictions in July 2020).
